# New Medical Journal

**Published:** 1858-03

**Authors:** 


NEW MEDICAL JOURNAL.—We have received the first (January) number of the *Pacific Medical and Surgical*

*Journal*, edited and published by John B. Trask, M.D., and David Wooster, M.D., of San Francisco, California. This new candidate for professional patronage is a monthly of forty-eight pages, and is now the only medical periodical published on the Pacific coast, the *California Medical Journal* having recently become extinct. We heartily wish it abundant success.

